# Comparison of Direct Laryngoscopy With McCoy Blade and BPL Video Laryngoscopy for Intubation in Patients Undergoing Elective Cervical Spine Surgery

**DOI:** 10.7759/cureus.74869

**Published:** 2024-11-30

**Authors:** Atit Kumar, Rakesh Bahadur Singh, Deepika Doneria, Usha Shukla, Mohammad Aleem Ansari

**Affiliations:** 1 Anesthesiology and Critical Care, Uttar Pradesh University of Medical Sciences, Etawah, IND

**Keywords:** airway management, bpl video laryngoscope, intubation, mccoy blade direct laryngoscope, patients with unstable cervical spine

## Abstract

Background: For patients having cervical spine instability, stabilization of the neck is crucial to prevent further damage to the spinal cord, which can make laryngoscopy challenging. Specialized tools like McCoy blade direct laryngoscope (Surgitech, India) and BPL video laryngoscope (BPL Medical Technologies Pvt. Ltd., India) enhance airway management in these groups of patients. This study compared the efficacy and safety of direct laryngoscopy with McCoy blade and BPL video laryngoscopy in cervical spine surgery patients.

Materials and methods: This randomized prospective comparative study was performed on 60 patients of ASA physical status I & II, either sex, 18 to 65 years of age, undergoing elective cervical spine surgery under general anesthesia. Patients were divided into groups B (BPL) and M (McCoy), with 30 patients in each group. After successful intubation, time for intubation, Modified Cormack Lehane grading, intubation difficulty score, ease of intubation, and hemodynamic parameters were recorded at numerous intervals.

Results: The mean duration for tracheal intubation was longer in Group B (36.30 ± 19.75 seconds) as compared to Group M (28.07 ± 10.19 seconds), which was statistically significant. The MCL grading, in Group B, was 76.67% and in Group M 46.67% of patients achieved MCL grade 1, which was statistically significant (p = 0.049), indicating better visualization of the vocal cords in Group B. Intubation difficulty score, in Group B, was 56.67% patients, and in Group M, only 23.33% patients achieved a score of 0 and it was also statically significant (p=0.042). 86.67% of patients in Group B and 60.00% of patients in Group M had ease of intubation scores 1, which were statistically significant (p = 0.040). All hemodynamic parameters were found statically insignificant in both groups.

Conclusions: This study revealed that BPL video laryngoscopy took more time for intubation but provided better vocal cord visualization and easier intubation, while McCoy blade intubation took less time for tracheal intubation and all hemodynamic parameters remained stable in both groups.

## Introduction

Endotracheal intubation is a procedure performed by medical professionals to secure a patient's airway and ensure oxygenation. It can be done using traditional laryngoscopes or video laryngoscopes to visualize the vocal cords and insert an endotracheal tube (ETT) into the trachea [1]. Conventional laryngoscopy requires significant upward and forward pressure on the handle to align the airway for visualizing the vocal cords. In contrast, video laryngoscopy typically involves less manipulation, potentially reducing hemodynamic stress during intubation [2].

Intubating the trachea is routine for anesthesiologists but can be challenging. Difficult airways may not be predictable and can emerge after induction of anesthesia. Patients undergoing cervical spine surgery often have limited neck movement, complicating intubation. To navigate this, McCoy blade direct laryngoscopy (Surgitech, India) and BPL video laryngoscopy (BPL Medical Technologies Pvt. Ltd., India) are commonly utilized [3]. Direct laryngoscopy with the McCoy blade lifts the epiglottis for clear visualization of the vocal cords, facilitating ETT insertion. However, in patients with limited neck movement, achieving a good view can be challenging, leading to longer intubation times and increased risks of airway damage and low oxygen levels [4].

The BPL VL-01 “non-channeled video laryngoscope” was employed in our study. The device is equipped with a two-megapixel complementary metal-oxide-semiconductor (CMOS) camera, a high-definition anti-fog lens, and an integrated LED light source. Additionally, it includes different-sized disposable blades that are securely fixed to the handle with a lock. The 4-inch anti-reflective LCD is of high resolution and has been designed to ensure maximum visibility. It is capable of 180-degree horizontal screen rotation and vertical movements, which enhances clinical assistance.

In this study, we have tried to compare BPL video laryngoscopy and direct laryngoscopy with McCoy blade for endotracheal intubation in terms of time duration for intubation as primary outcome, Modified Cormack Lehane (MCL) grading, intubation difficulty score (IDS), ease of intubation, success rate of intubation, and hemodynamic parameters as secondary outcomes. The goal was to determine the most effective technique for managing the airway in this challenging group of patients.

## Materials and methods

This study was carried out in the Department of Anesthesiology, from February 2024 to September 2024, after approval from the Institutional Ethics Committee of Uttar Pradesh University of Medical Sciences, Saifai-Etawah, Uttar Pradesh, India. We have conducted a prospective randomized comparative study on 60 patients with cervical instability undergoing elective cervical spine surgery under general anesthesia (GA), having ages of 18 to 65, with ASA physical status I and II, and had a Mallampati Score (MPS) grade of I or II. Patients with a mouth opening of <3 cm, basal metabolic index (BMI) >35 kg/m^2^, risk of aspiration were excluded.

According to the study of Panwar et al. [5], the mean difference in the mean duration of tracheal intubation (sec.) in the indirect (18.50) and direct group (11.76 sec.) was 6.74, and the average variance (σ2) was 7.85. The formula used to calculate sample size, n = 2 (Zα/2 + Z [1-β])2 × σ2/(μ1−μ2)2, where n = Sample size, Zα/2 = Level of significance, Z [1-β] = Power of study, σ2 = Average variance, μ1= mean duration of tracheal intubation in BPL video laryngoscopy group, and μ2 = mean duration of tracheal intubation in direct laryngoscopy with McCoy blade group. Each group's 90% power (Z [1-β]) = 1.28 and 0.05 level significance (Zα/2 = 1.96), respectively, were 28.48. Hence, in the round figure, we enrolled a minimum of 30 patients in each group.

A total of 60 patients were assessed for eligibility and included in the study with no exclusions. Each patient underwent a comprehensive pre-anesthetic check-up and pre-operative airway assessment conducted by the same anesthesiologist who performed all laryngoscopies. The assessment included inspection of the neck for signs of deformity, tenderness, or instability in the cervical spine; MPS grading; evaluation of mouth opening; and measurement of body mass index (BMI). Randomization was carried out using a computer-generated random number table to ensure baseline comparability between the groups. Allocation concealment was maintained by using sequentially numbered, opaque sealed envelopes, ensuring that the assignment of participants to Group B (B patients received BPL video laryngoscopy) and Group M (patients received direct laryngoscopy with McCoy blade), each consisting of 30 patients, was unbiased and unaffected by the enrolling personnel. All 60 patients completed the study without any dropouts, and the follow-up period was consistent for all participants. Consequently, all 60 patients were included in the final analysis.

In the pre-operating room, baseline hemodynamic parameters heart rate (HR), systolic blood pressure (SBP), diastolic blood pressure (DBP), mean arterial pressure (MAP), and peripheral oxygen saturation (SpO_2_) were recorded. The patient was transferred to the operating room, where peripheral vascular access was established using an 18-gauge intravenous cannula in all cases. A ringer lactate infusion was started at a rate of 15 mL per kilogram of body weight. Both groups of patients received intravenous injections of Ondansetron (0.1 mg/kg), ranitidine (2 mg/kg), Glycopyrrolate (5 mcg/kg), Midazolam (0.02 mg/kg), and Fentanyl (2 µg/kg) as premedication five minutes before the anesthesia was induced. Additionally, all patients were preoxygenated with 100% oxygen through a face mask for three minutes. Propofol injection (2 mg/kg) was administered as an induction agent, followed by succinylcholine injection (2 mg/kg) as a muscle relaxant. Subsequently, laryngoscopy was done using a direct laryngoscope with McCoy blades in group M, and with a BPL video-laryngoscope in group B. The laryngoscopy and intubation procedures were conducted by an “anesthesiologist” participating in this study, with cervical spine stabilization techniques such as Manual in-line stabilization (MILS) of the neck. It was performed by an experienced anesthesiologist, who stood on the right side of the supine patient, facing the patient’s head. The anesthesiologist's hands were placed on either side of the neck, with fingers pressing on the mastoid processes, to maintain neutral alignment and immobilize the head and neck. The tracheal intubation time (in sec) “from handling of intubating device and insertion into the mouth until the tracheal tube is properly placed between the vocal cords” was recorded by an independent individual who was not involved in the study. We also recorded MCL grading, IDS, ease of intubation, the number of intubation attempts, and hemodynamic parameters variations, including HR, SBP, DBP, MAP, and SpO2, at different points of time, i.e., immediately after intubation, one minute, five minutes, and 10 min. All patient’s airways are secured by using polyvinyl chloride (PVC) endotracheal tubes with an internal diameter ranging from 7.0 to 8.5 mm according to patients. All patients were given personalized care with appropriate fluid management and continuous monitoring to ensure their well-being during intraoperative procedures.

Ease of intubation

Grade I - Tracheal intubation without maneuver (Easy)

Grade II - Tracheal intubation with maneuver (Satisfactory)

Grade III - Tracheal intubation not even with maneuver (Difficulty) [5].

Intubation difficulty score (IDS)

It is a numerical scoring system designed to evaluate the difficulty of intubation in patients (Table 1) [6].

**Table 1:** Intubation difficulty score IDS Score and Degree of Difficulty: 0 = Easy, >0 to <5 = Slight Difficulty, >5 = Moderate to Major Difficult

Parameters	Score	Calculating methods
Number of attempts >1	N1	Every additional attempt adds 1 point
Number of operators >1	N2	Every operator adds 1 point
Number of alternative techniques >1	N3	Each alternative technique adds 1 point. e.g. intubation stylet, video laryngoscope, and/or fiber optic bronchoscope.
Cormack grade - 1	N4	Apply Cormack grade for 1^st^ oral attempts
Lifting force required normal, increased	N5=0, N5=1	NA/-
Laryngeal pressure not applied, applied	N6=0, N6=1	NA/-
Vocal cord mobility abduction, adduction	N7=0, N7=1	NA/-
Total IDS score = sum of scores	N1 - N7	NA/-

The statistical analysis of data was performed with SPSS version 21.0 (IBM Corp., Armonk, NY). The data were presented in the form of mean (standard deviation) and percentage (%). The chi-square test was used to compare categorical variables between groups. A p-value of < 0.05 was considered statistically significant.

## Results

**Figure 1:**
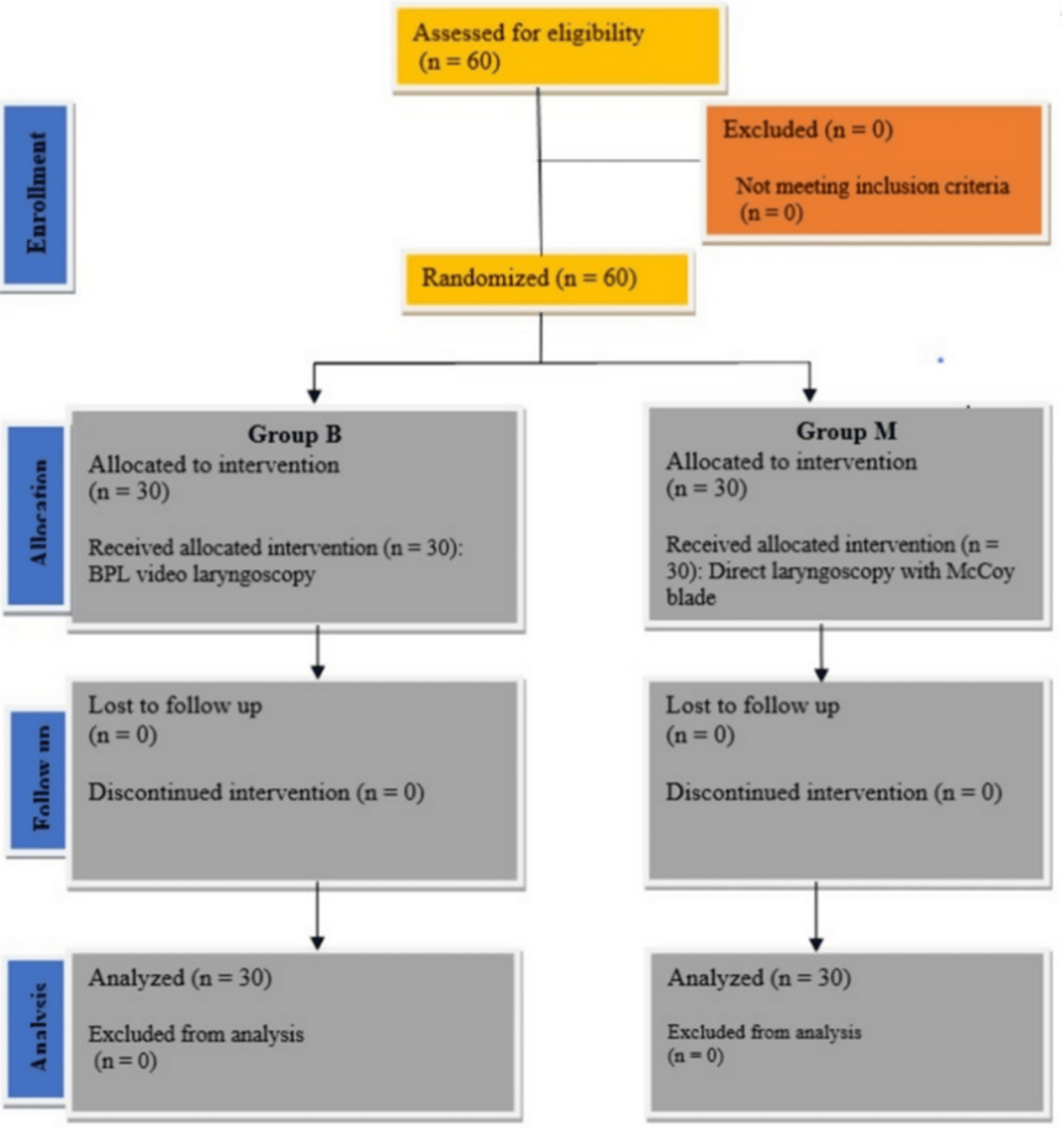
Consolidated Standards of Reporting Trials (CONSORT) flow diagram.

Sixty patients scheduled to undergo cervical spine surgery under GA were randomly divided into two groups of 30 patients each (Figure 1), no patients were excluded from this study. The demographic variables (age, sex, BMI) and ASA physical status classification were statistically not significant between study groups (p < 0.05) (Table 2).

**Table 2:** Comparison of the demographic profile between the two groups, t-test, and chi-square test. Group B = BPL video laryngoscopy group and Group M = McCoy blade direct laryngoscopy group.

Variables		Group B (n=30)	Group M (n=30)	P-value
Age distribution	Mean ± SD	43.23 ± 13.24	40.27 ± 16.05	p=0.438
Gender distribution	Male	83.33% (n=25)	86.67% (n=26)	p=0.718
	Female	16.67% (n=5)	13.33% (n=4)	
BMI distribution (kg/m^2^)	Mean ± SD	21.01 ± 1.36	21.08 ± 1.46	p=0.855
ASA class status	I	33.33% (n=10)	40.00% (n=12)	p=0.789
	II	66.67% (n=20)	60.00% (n=18)	

In our study, the mean duration of tracheal intubation (in seconds) was 36.30 ± 19.75 in group B and 28.07 ± 10.19 in group M. There is a significant difference in the duration of tracheal intubation between groups; therefore, group B has a longer average duration of intubation compared to group M for elective cervical spine procedures (Table 3).

**Table 3:** Comparison of mean duration of the tracheal intubation (in sec) between group B and group M. Group B = BPL video laryngoscopy group and Group M = McCoy blade direct laryngoscopy group.

	Group B	Group M	t	P-value
	Mean ± SD	Mean ± SD		
Duration of the tracheal intubation (in seconds)	36.30 ± 19.75	28.07 ± 10.19	2.03	0.047

MCL grading showed a significant difference (p = 0.049) between Group B (76.67% Grade 1) and Group M (46.67% Grade 1), The chi-square test showed a significant difference in MCL grading between the groups (chi-square = 6, p = 0.049) means Group B had better visualization of the vocal cords compared to Group M (Table 4).

**Table 4:** Comparison modified Cormack Lehane grading between two groups. Group B = BPL video laryngoscopy group and Group M = McCoy blade direct laryngoscopy group.

		Group B	Group M	Chi Sq.	P-value
		n (%)	n (%)		
Modified Cormack Lehane grading	1	23 (76.67)	14 (46.67)	6.00	0.049
	2A	41(3.33)	7 (23.33)		
	2B	3 (10.00)	9 (30.00)		
	3A	0 (0.00)	0 (0.00)		
	3B	0 (0.00)	0 (0.00)		
	4	0 (0.00)	0 (0.00)		

In Group B, 56.67% had a score of 0, while in Group M, only 23.33% achieved the same score. The chi-square test revealed a significant difference in IDS between the groups (chi-square = 8.22, p = 0.042). This indicates that Group B had a significantly lower proportion of subjects with high scores, demonstrating less difficult intubation in Group B compared to Group M (Table 5).

**Table 5:** Comparison of intubation difficulty score (IDS) between group B and group M. Group B = BPL video laryngoscopy group and Group M = McCoy blade direct laryngoscopy group.

		Group B	Group M	Chi Sq.	P-value
		n (%)	n (%)		
Intubation difficulty score (IDS)	0	17 (56.67)	7 (23.33)	8.22	0.042
	1-5	13 (43.33)	23 (76.67)		
	>5	0 (0.00)	0 (0.00)		

Ease of intubation score was observed as grade I: 86.67% in group B and 60.0% in group M, and as grade II: 13.33% patients in group B and 40.0% patients of group M required backward, upward, and rightward pressure (BURP) on the larynx during intubation. Based on the data, both groups were statically significant (chi sq. 4.18, p 0.04) (Table 6).

**Table 6:** Comparison of ease of intubation score between two groups. Group B = BPL video laryngoscopy group and Group M = McCoy blade direct laryngoscopy group.

		Group B	Group M	Chi Sq.	P-value
		n (%)	n (%)		
Ease of intubation score	Grade I	26 (86.67)	18 (60.00)	4.18	0.040
	Grade II	4 (13.33)	12 (40.00)		
	Grade III	0 (0.00)	0 (0.00)		

All patients were successfully intubated using the designated laryngoscope in both groups. The first attempt success rate was 96.67% (n=29) in Group B and 86.67% (n=26) in Group M, rest patients in both groups required a second attempt, indicating a higher level of success in Group B during the first intubation attempts (Table 7). All the hemodynamic parameters (HR, SBP, DBP, MAP) noted no statistically significant difference between the two groups (Table 8).

**Table 7:** Number of attempt(s) for endotracheal intubation.

		Group B	Group M	Chi Sq.	P-value
		n (%)	n (%)		
No. of attempts	1^st^	29 (96.67)	26 (86.67)	0.87	0.350
	2^nd^	1 (3.33)	4 (13.33)		

**Table 8:** Hemodynamic parameters response after intubation.

Parameters		Group B	Group M	t	P-value
		Mean± SD	Mean± SD		
Heart rate	Baseline	79.83±9.11	79.60±10.91	0.09	0.929
	Just after intubation	97.47±13.20	102.13±13.51	0.10	0.323
	At 1 min	92.27±13.39	95.53±13.21	0.50	0.616
	At 5 min	89.70±10.96	87.20±11.42	1.21	0.231
	At 10 min	87.23±9.87	83.43±11.63	1.36	0.178
Systolic BP	Baseline	123.87±10.48	120.20±9.97	1.39	0.170
	Just after intubation	130.80±11.65	132.40±19.58	1.30	0.499
	At 1 min.	125.07±10.10	128.47±8.23	1.93	0.258
	At 5 min	123.13±9.96	124.60±7.58	1.07	0.291
	At 10 min	123.93±9.93	122.60±8.82	1.79	0.079
Diastolic BP	Baseline	83.13±7.10	80.33±8.50	1.38	0.171
	Just after intubation	89.13±6.90	88.93±6.25	0.12	0.907
	At 1 min	86.80±6.49	84.60±6.31	1.33	0.188
	At 5 min	84.2±6.50	81.67±5.94	1.60	0.116
	At 10 min	83.47±7.84	80.40±7.45	1.55	0.126
Mean arterial pressure	Baseline	97.13±8.73	93.93±9.29	-1.38	0.174
	Just after intubation	103.03±8.33	104.80±6.59	-0.64	0.527
	At 1 min.	100.70±6.82	99.47±6.18	-1.92	0.059
	At 5 min	94.60±6.15	95.83±5.54	-1.83	0.072
	At 10 min	93.80±8.16	94.40±7.55	-1.68	0.099

## Discussion

In our research, we found that the duration of tracheal intubation (Table 3), was 36.30 ± 19.75 seconds in group B and 28.07 ± 10.19 seconds in group M, which was statistically significant (p-value = 0.047), therefore group B had a longer average duration of intubation compared to group M. Similarly, Panwar et al. [5] found that the indirect group (video laryngoscope) had a mean duration of 18.50 ± 11.25 seconds, whereas the direct group (Macintosh group) had 11.76 ± 4.44 seconds, this difference was statistically significant, showing that indirect intubation took longer time than the direct group. Our result showed discordance with Rohini et al. [7] mean duration of intubation was comparable between the C MAC group (26.00 sec) and the McCoy group (26.55 sec) and statistically insignificant.

The MCL grading (Table 4) revealed significant differences between the groups (p = 0.049), indicating that group B had better visualization of the vocal cords compared to group M. In Group B, 76.67% of patients were classified as Grade 1, 13.33% as Grade 2A, 10.00% as Grade 2B, in contrast, Group M had 46.67% of patients classified as Grade 1, 23.33% as Grade 2A, 30.00% as Grade 2B. However, more Group M patients were Grade 2 (2A and 2B), suggesting slightly difficult laryngoscopic views. This considerable difference in MCL grading showed that BPL video laryngoscopy helps to improve glottic visibility. In concordance to our study Panwar et al. [5], showed that higher MCL grades in the direct group as compared to the indirect group which was statistically significant. Our study goes in favor of Kumari et al. [8] who found MCL grade 1 had a larger proportion of the C-MAC D-blade group in comparison to the McCoy group, and it was statistically significant.

Our study shows IDS (Table 5), in group B, 56.67% patients of scored 0, 43.33% of score 1-5, 0.00% of score >5, while group M had 23.33% patients of scored 0, 73.33% of score 1-5, 0.00% of score >5. A significant difference in IDS was noted between the groups (p = 0.042). This indicates that a higher proportion of subjects in group B as compared to group M experienced easier intubations (IDS=0). This finding aligns with the study of Panwar et al. [5] and Manikanta et al. [9] which were statistically significant.

Ease of intubation score (Table 6) was observed as grade I: 86.67% in group B and 60.0% in group M, which means any laryngeal manipulation was not required during intubation, and as grade II:13.33% patients in group B and 40.0% patients of group M required BURP on the larynx during intubation. None of the patients (0.00%) in both groups had ease of intubation grade III. Based on the data, both groups were statically significant (chi sq. 4.18, p 0.04), suggesting that intubation was generally easier in group B than in group M. Similar to our study, Shukla et al. [10] observed that the maximum number of patients in both groups required no assistance during intubation, only 11.11% of patients in group A (Macintosh blade) and 19.44% of patients in group B (BPL VL-02) needed BURP on the larynx during intubation and it was statistically significant (p=0.030). In discordance with our study, Panwar et al. [5] found a significant difference in ease of intubation (P<0.001) between the groups, and most of the patients in the glidescope group required laryngeal pressure.

The number of attempts for intubation compared in both groups (Table 7), in group B, 96.67% of patients, and in group M 86.67% were successfully intubated on the first attempt. Only 3.33% of patients in Group B and 13.33% in Group M required a second intubation attempt. The chi-square test was 0.87 with a p-value of 0.350, indicating that both groups had comparable success rates in terms of intubation attempts, but the difference was not statistically significant. A similar result was also obtained by Cavus et al. [11] and Panwar et al. [5] which were statistically insignificant.

The hemodynamic parameters (Table 8) were comparable in both groups and statistically nonsignificant difference was noted, however, it was observed that heart rate and blood pressure were more stable in Group B, suggesting that BPL video laryngoscopy may provide a slight advantage in maintaining heart rate stability during and after intubation. A similar result was reported by Sabrya et al. [12] as McCoy patients had higher hemodynamic parameter changes than C-MAC D-blade patients until four minutes after intubation.

This study has some limitations. First, the anesthesiologist’s assessment of the glottis view could not be blinded, which may introduce observer bias. Second, the sample size was relatively small, a larger sample size could yield more precise results. Lastly, if tracheal intubation had been performed by more experienced anesthesiologists, it might have taken less time for intubation.

## Conclusions

The BPL video laryngoscope group exhibited a longer intubation time compared to direct laryngoscopy with the McCoy blade. However, it provided superior visibility and facilitated easier insertion of the ETT, making it a more favorable choice for elective cervical spine surgeries. Hemodynamic responses were similar between the two techniques, suggesting no significant differences in cardiovascular stability during and after intubation. Given the elective nature of these procedures, the BPL video laryngoscope may be considered the preferred option due to its enhanced visualization and ease of use.
